# Severe Symptomatic Hypocalcemia from HIV Related Hypoparathyroidism

**DOI:** 10.1155/2018/8270936

**Published:** 2018-09-25

**Authors:** Sartaj Sandhu, Akshata Desai, Manav Batra, Robin Girdhar, Kaushik Chatterjee, E. Helen Kemp, Antoine Makdissi, Ajay Chaudhuri

**Affiliations:** ^1^Advocare DelGiorno Endocrinology, Sewell, New Jersey, USA; ^2^Department of Endocrinology, Diabetes and Metabolism, State University of New York, Buffalo, New York, USA; ^3^Department of Oncology and Metabolism, University of Sheffield, Sheffield, UK

## Abstract

We report the case of a 54-year-old Caucasian female who presented with a two-year history of persistent hypocalcemia requiring multiple hospitalizations. Her medical history was significant for HIV diagnosed four years ago. She denied any history of prior neck surgery or radiation. Her vital signs were stable with an unremarkable physical exam. Pertinent medications included calcium carbonate, vitamin D3, calcitriol, efavirenz, emtricitabine, tenofovir disoproxil, hydrochlorothiazide, and inhaled budesonide/formoterol. Laboratory testing showed total calcium of 5.7 mg/dL (normal range: 8.4-10.2 mg/dL), ionized calcium of 2.7 mg/dL (normal range: 4.5-5.5 mg/dL), serum phosphate of 6.3 mg/dL (normal range: 2.7-4.5 mg/dL), and intact PTH of 7.6 pg/mL (normal range: 15-65 pg/mL). She was diagnosed with primary hypoparathyroidism. Anti-calcium-sensing receptor antibodies and NALP5 antibodies were tested and found to be negative. During subsequent clinic visits, doses of calcium supplements and calcitriol were titrated. Last corrected serum calcium level was 9.18 mg/dL. She was subsequently lost to follow-up. This case gives insight into severe symptomatic hypocalcemia from primary hypoparathyroidism attributed to HIV infection. We suggest that calcium levels should be closely monitored in patients with HIV infection.

## 1. Introduction

Endocrine involvement is frequent in HIV infected patients, although it rarely involves the parathyroid glands [1]. Hypocalcemia is an infrequent phenomenon in HIV infection and it is mostly attributed to Vitamin D deficiency, hypoalbuminemia, or pharmacotherapy. We report a rare case of severe symptomatic hypocalcemia from primary hypoparathyroidism attributed to HIV infection.

## 2. Case Presentation

A 54-year-old Caucasian female presented to our clinic with a two-year history of persistent hypocalcemia requiring multiple hospitalizations. Her symptoms included muscle cramps, tingling and perioral paresthesias. Her medical history was significant for HIV diagnosed four years ago, gastric bypass surgery done 15 years ago, hypertension, and COPD. She denied any history of prior neck surgery or radiation. She denied any history of hearing loss. She had no family history of autoimmune disease.

Her vital signs were stable with an unremarkable physical exam. Chvostek's and Trousseau's signs were negative. Pertinent medications included calcium carbonate, vitamin D3, calcitriol, atripla (efavirenz/emtricitabine/tenofovir disoproxil), hydrochlorothiazide, and inhaled budesonide/formoterol.

Laboratory testing showed total calcium of 5.7 mg/dL (normal range: 8.4-10.2 mg/dL), serum albumin 3.9 mg/dL, ionized calcium 2.7 mg/dL (normal range: 4.5-5.5 mg/dL), serum magnesium 1.7 mg/dL (normal range: 1.7-2.7 mg/dL), serum phosphate 6.3 mg/dL (normal range: 2.7-4.5 mg/dL), and intact PTH 7.6 pg/mL (normal range: 15-65 pg/mL). She had normal 25-hydroxy vitamin D 32 ng/mL (normal range: 30-100 ng/mL), 1,25 dihydroxy vitamin D 23 pg/mL (normal range: 18-72 pg/mL), TSH 1.2 *μ*IU/L (normal range: 0.40-4.5 *μ*IU/L), and creatinine 0.98 mg/dL (normal range: 0.5 -1.1 mg/dL). Absolute CD4 count was 629 cells/*μ*L (normal range: 185-2273 cells/*μ*L) with undetectable HIV-1 RNA viral load.

She was diagnosed with primary hypoparathyroidism. A serum sample was tested for anti-calcium sensing receptor (CaSR) antibodies [2] and NALP5 antibodies [2] to rule out autoimmune hypoparathyroidism and it was found to be negative; the CaSR antibody index was 1.09 (normal range: 0.57-1.38; upper limit of normal, 1.73) and the NALP5 antibody index was 1.12 (normal range: 0.62-1.93; upper limit of normal, 2.17). During subsequent clinic visits, doses of calcium supplements and calcitriol were titrated and she was started on magnesium oxide. She required calcium carbonate 2500 mg three times per day, calcium citrate 1900 mg twice per day, and calcitriol 0.5 mcg three times per day (Table 1). Her last corrected serum calcium level was 9.18 mg/dL. She was considered for treatment with recombinant human PTH but subsequently she was lost to follow-up.

## 3. Discussion

Hypocalcemia is defined as low albumin corrected total serum calcium or low ionized serum calcium levels. It is a relatively common condition. Broadly, the causes of hypocalcemia are divided into disorders associated with low or high PTH levels, drugs, and hypomagnesemia [3], as shown in Table 2.

Increased prevalence of hypocalcemia has been reported in HIV positive individuals, but it was mostly related to vitamin D deficiency [4]. A retrospective review reported greater frequency of parathyroid hyperplasia in autopsy specimens of HIV infected African American patients, which could be result of high prevalence of vitamin D deficiency in HIV infected patients [5].

Other causes of hypocalcemia in HIV infected patients are attributed to hypoalbuminemia [6], medications like foscarnet [7] and Fanconi's syndrome related to antiretroviral medications [8]. Tenofovir can rarely cause hypocalcemia as part of Fanconi's syndrome, along with hypophosphatemia and normal or slightly elevated PTH levels [9].

Our patient had hypocalcemia, hyperphosphatemia with low PTH levels, consistent with primary hypoparathyroidism. With the absence of neck surgery and an unlikely autoimmune etiology (negative antibody testing, late onset, absence of family history, hearing loss, and associated conditions), HIV infection was considered as the principal etiology causing hypoparathyroidism leading to hypocalcemia. This has been very rarely reported in literature [10].

The cause of hypoparathyroidism in HIV infected individuals is thought to be related to impaired parathyroid hormone release and altered parathyroid function [11, 12]. Mechanisms explaining hypoparathyroidism in HIV infected patients are not well described. There is suggestion of expression of a CD4 like molecule by the parathyroid cells making them a potential target of HIV, leading to impaired PTH secretion [13].

Further, our patient was unique and challenging to manage due to requirement of high doses of calcium, calcitriol, and Vitamin D. It may be in part due to previous history of gastric bypass surgery which may impair calcium and Vitamin D absorption.

## 4. Conclusion

Although rare, primary hypoparathyroidism should be considered in the differential diagnosis of hypocalcemia in HIV infected patients. We suggest that calcium levels, PTH levels, and 25(OH) vitamin D levels should be monitored regularly in those patients.

## Figures and Tables

**Table 1:** Clinical course showing laboratory studies and treatment regimen.

**Date**	**Corrected total calcium (normal range, 8.4–10.2 mg/dL)**	**Ionized calcium (normal range, 4.5-5.5 mg/dL)**	**Serum phosphate (normal range, 2.7–4.5 mg/dL)**	**Medications**
11/2012	6.9	3.0	5.9	Calcitriol 0.25 mcg daily Total elemental calcium 1440 mg daily (as calcium carbonate 3600 mg daily in divided doses) Vitamin D 50,000 units weekly

12/2012	7.0	3.5	5.9	Calcitriol 0.25 mcg twice daily Total elemental calcium 2400 mg daily (as calcium carbonate 6000 mg daily in divided doses) Vitamin D 50,000 units weekly

3/2013	7.2	3.5	5.7	Calcitriol 0.25 mcg three times daily Total elemental calcium 1800 mg daily (as calcium carbonate 4500 mg daily in divided doses) Vitamin D 50,000 units weekly

6/2013	7.0	3.2	5.4	Calcitriol 0.5 mcg twice daily Total elemental calcium 2000 mg daily (as calcium carbonate 5000 mg daily in divided doses) Vitamin D 50,000 units weekly

8/2013	7.9	-	-	Calcitriol 0.5 mcg three times daily Total elemental calcium 3600 mg daily (as calcium carbonate 7500 mg daily and calcium citrate 2850 mg daily in divided doses) Vitamin D 50,000 units weekly

11/2013	9.2	4.8	-	Calcitriol 0.5 mcg three times daily Total elemental calcium 3800 mg daily (as calcium carbonate 7500 mg daily and calcium citrate 3800 mg daily in divided doses) Vitamin D 50,000 units weekly

**Table 2:** Pathogenesis and differential diagnosis of hypocalcemia.

**Hypocalcemia associated with**	**Disorders**
Low PTH	Abnormal PTH synthesis; Abnormal parathyroid gland development; Post-surgical hypoparathyroidism; Autoimmune polyglandular syndrome type 1; Activating mutations of the calcium-sensing receptor; Infiltration of parathyroid gland; Radiation-induced hypoparathyroidism; HIV infection; Hungry bone syndrome

High PTH	Vitamin D deficiency or resistance; PTH resistance (pseudohypoparathyroidism); Loss of calcium in circulation (tumor lysis syndrome, acute pancreatitis, sepsis, osteoblastic metastases, hyperphosphatemia); Renal disease

Drugs	Inhibitors of bone resorption (bisphosphonates, calcitonin, denosumab); Cinacalcet; Calcium chelators (EDTA, citrate, phosphate); Foscarnet; Phenytoin; Fluoride poisoning

Disorders of magnesium metabolism	Hypomagnesemia causing functional hypoparathyroidism
